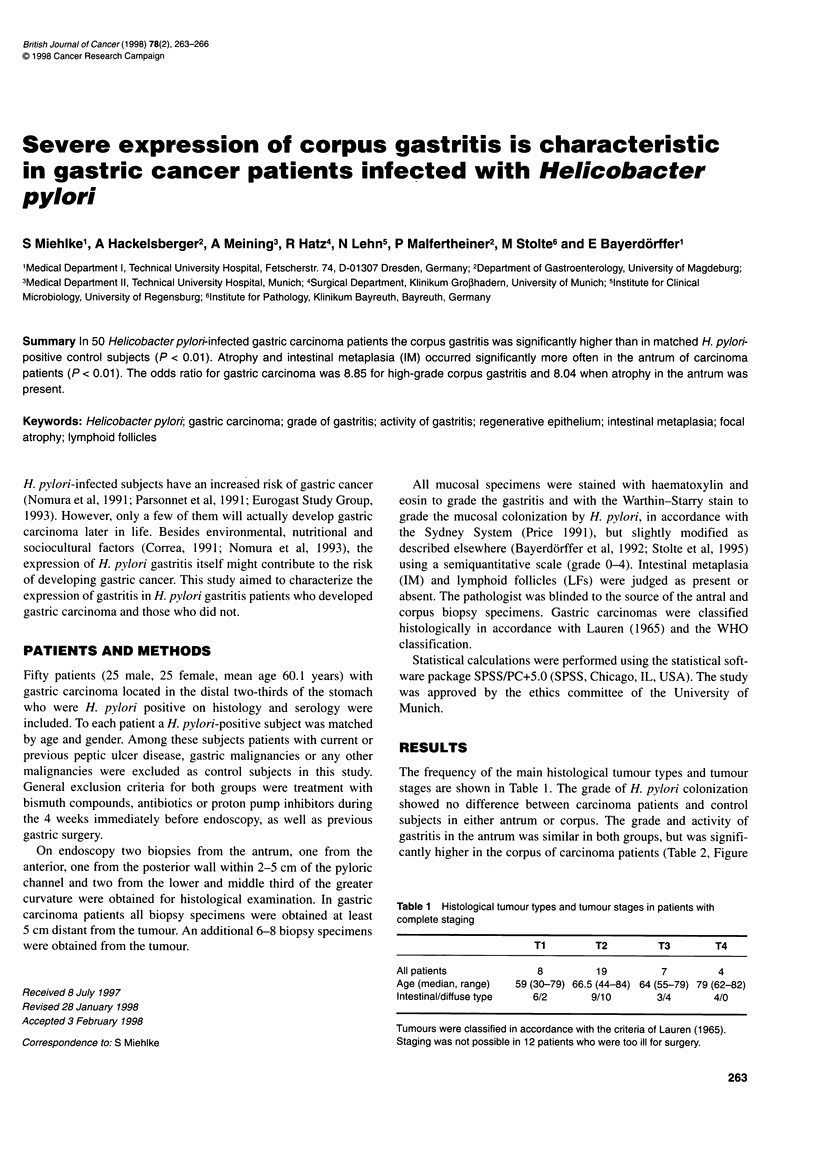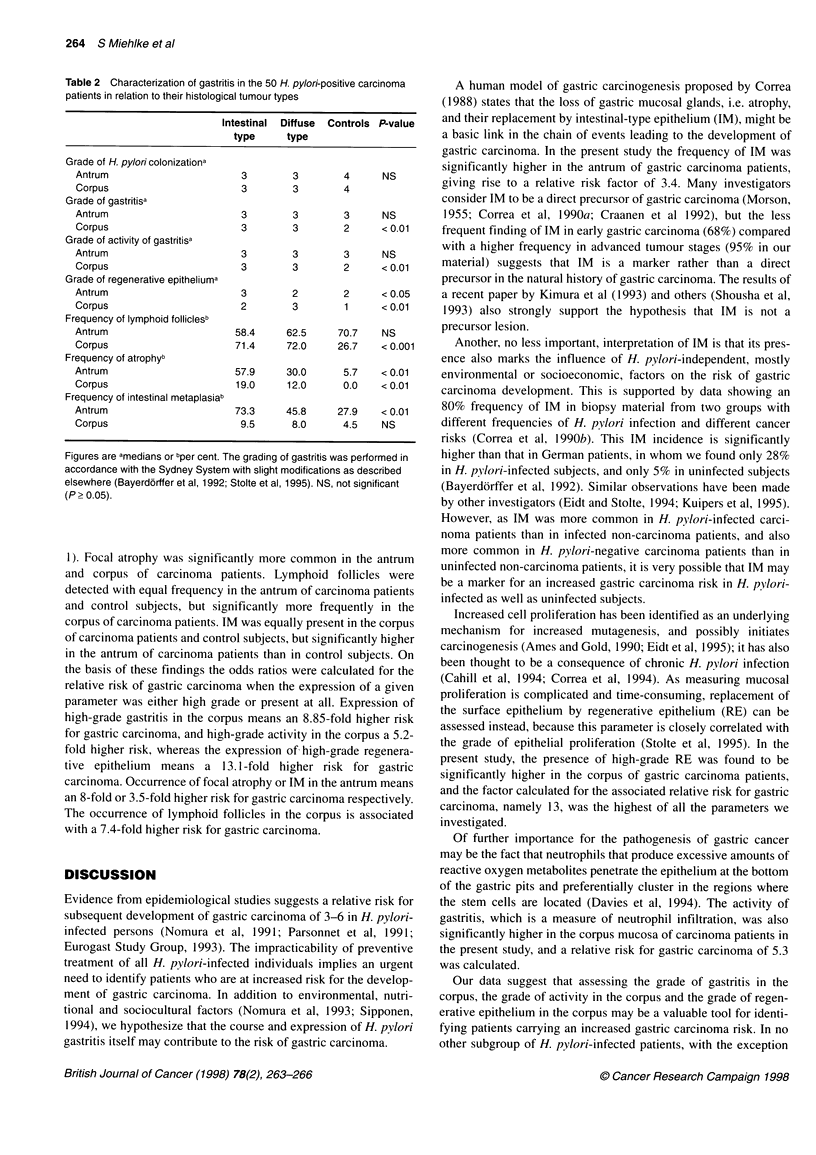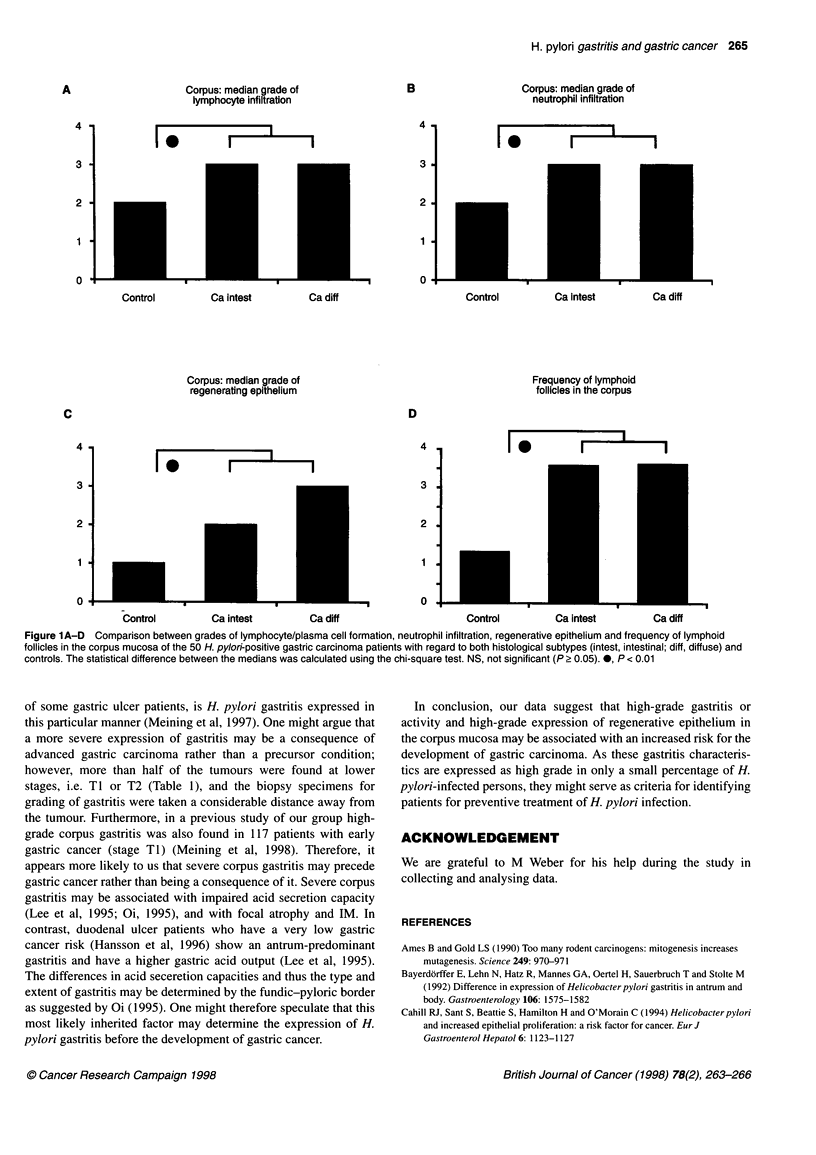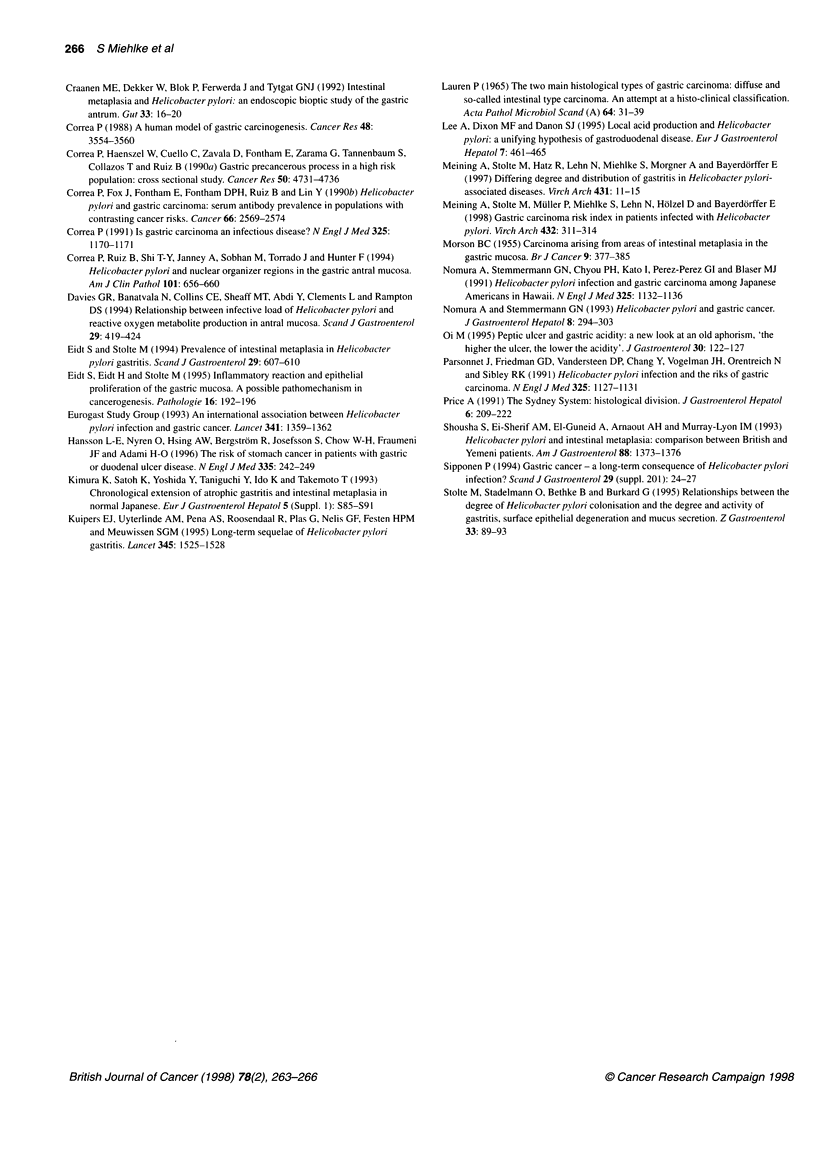# Severe expression of corpus gastritis is characteristic in gastric cancer patients infected with Helicobacter pylori.

**DOI:** 10.1038/bjc.1998.475

**Published:** 1998-07

**Authors:** S. Miehlke, A. Hackelsberger, A. Meining, R. Hatz, N. Lehn, P. Malfertheiner, M. Stolte, E. Bayerdörffer

**Affiliations:** Medical Department I, Technical University Hospital, Dresden, Germany.

## Abstract

In 50 Helicobacter pylori-infected gastric carcinoma patients the corpus gastritis was significantly higher than in matched H. pylori-positive control subjects (P < 0.01). Atrophy and intestinal metaplasia (IM) occurred significantly more often in the antrum of carcinoma patients (P < 0.01). The odds ratio for gastric carcinoma was 8.85 for high-grade corpus gastritis and 8.04 when atrophy in the antrum was present.


British Journal of Cancer (1998) 78(2), 263–266
© 1998 Cancer Research Campaign

### Severe expression of corpus gastritis is characteristic in gastric cancer patients infected with *Helicobacter pylori*

**S Miehlke[1], A Hackelsberger[2], A Meining[3], R Hatz[4], N Lehn[5], P Malfertheiner[2], M Stolte[6] and E Bayerdörffer[1]**

[1]Medical Department I, Technical University Hospital, Fetscherstr. 74, D-01307 Dresden, Germany; [2]Department of Gastroenterology, University of Magdeburg; [3]Medical Department II, Technical University Hospital, Munich; [4]Surgical Department, Klinikum Großhadern, University of Munich; [5]Institute for Clinical Microbiology, University of Regensburg; [6]Institute for Pathology, Klinikum Bayreuth, Bayreuth, Germany

**Summary** In 50 *Helicobacter pylori*-infected gastric carcinoma patients the corpus gastritis was significantly higher than in matched *H. pylori*-positive control subjects ($P < 0.01$). Atrophy and intestinal metaplasia (IM) occurred significantly more often in the antrum of carcinoma patients ($P < 0.01$). The odds ratio for gastric carcinoma was 8.85 for high-grade corpus gastritis and 8.04 when atrophy in the antrum was present.

**Keywords:** *Helicobacter pylori*; gastric carcinoma; grade of gastritis; activity of gastritis; regenerative epithelium; intestinal metaplasia; focal atrophy; lymphoid follicles

*H. pylori*-infected subjects have an increased risk of gastric cancer (Nomura et al, 1991; Parsonnet et al, 1991; Eurogast Study Group, 1993). However, only a few of them will actually develop gastric carcinoma later in life. Besides environmental, nutritional and sociocultural factors (Correa, 1991; Nomura et al, 1993), the expression of *H. pylori* gastritis itself might contribute to the risk of developing gastric cancer. This study aimed to characterize the expression of gastritis in *H. pylori* gastritis patients who developed gastric carcinoma and those who did not.

### PATIENTS AND METHODS

Fifty patients (25 male, 25 female, mean age 60.1 years) with gastric carcinoma located in the distal two-thirds of the stomach who were *H. pylori* positive on histology and serology were included. To each patient a *H. pylori*-positive subject was matched by age and gender. Among these subjects patients with current or previous peptic ulcer disease, gastric malignancies or any other malignancies were excluded as control subjects in this study. General exclusion criteria for both groups were treatment with bismuth compounds, antibiotics or proton pump inhibitors during the 4 weeks immediately before endoscopy, as well as previous gastric surgery.

On endoscopy two biopsies from the antrum, one from the anterior, one from the posterior wall within 2–5 cm of the pyloric channel and two from the lower and middle third of the greater curvature were obtained for histological examination. In gastric carcinoma patients all biopsy specimens were obtained at least 5 cm distant from the tumour. An additional 6–8 biopsy specimens were obtained from the tumour.

*Received 8 July 1997*
*Revised 28 January 1998*
*Accepted 3 February 1998*

*Correspondence to:* S Miehlke

All mucosal specimens were stained with haematoxylin and eosin to grade the gastritis and with the Warthin–Starry stain to grade the mucosal colonization by *H. pylori*, in accordance with the Sydney System (Price 1991), but slightly modified as described elsewhere (Bayerdörffer et al, 1992; Stolte et al, 1995) using a semiquantitative scale (grade 0–4). Intestinal metaplasia (IM) and lymphoid follicles (LFs) were judged as present or absent. The pathologist was blinded to the source of the antral and corpus biopsy specimens. Gastric carcinomas were classified histologically in accordance with Lauren (1965) and the WHO classification.

Statistical calculations were performed using the statistical software package SPSS/PC+5.0 (SPSS, Chicago, IL, USA). The study was approved by the ethics committee of the University of Munich.

### RESULTS

The frequency of the main histological tumour types and tumour stages are shown in Table 1. The grade of *H. pylori* colonization showed no difference between carcinoma patients and control subjects in either antrum or corpus. The grade and activity of gastritis in the antrum was similar in both groups, but was significantly higher in the corpus of carcinoma patients (Table 2, Figure

**Table 1** Histological tumour types and tumour stages in patients with complete staging

| | T1 | T2 | T3 | T4 |
|---|---|---|---|---|
| All patients | 8 | 19 | 7 | 4 |
| Age (median, range) | 59 (30–79) | 66.5 (44–84) | 64 (55–79) | 79 (62–82) |
| Intestinal/diffuse type | 6/2 | 9/10 | 3/4 | 4/0 |

Tumours were classified in accordance with the criteria of Lauren (1965). Staging was not possible in 12 patients who were too ill for surgery.

**Table 2** Characterization of gastritis in the 50 *H. pylori*-positive carcinoma patients in relation to their histological tumour types

| | Intestinal type | Diffuse type | Controls | *P*-value |
|---|---|---|---|---|
| Grade of *H. pylori* colonization[a] | | | | |
| Antrum | 3 | 3 | 4 | NS |
| Corpus | 3 | 3 | 4 | |
| Grade of gastritis[a] | | | | |
| Antrum | 3 | 3 | 3 | NS |
| Corpus | 3 | 3 | 2 | < 0.01 |
| Grade of activity of gastritis[a] | | | | |
| Antrum | 3 | 3 | 3 | NS |
| Corpus | 3 | 3 | 2 | < 0.01 |
| Grade of regenerative epithelium[a] | | | | |
| Antrum | 3 | 2 | 2 | < 0.05 |
| Corpus | 2 | 3 | 1 | < 0.01 |
| Frequency of lymphoid follicles[b] | | | | |
| Antrum | 58.4 | 62.5 | 70.7 | NS |
| Corpus | 71.4 | 72.0 | 26.7 | < 0.001 |
| Frequency of atrophy[b] | | | | |
| Antrum | 57.9 | 30.0 | 5.7 | < 0.01 |
| Corpus | 19.0 | 12.0 | 0.0 | < 0.01 |
| Frequency of intestinal metaplasia[b] | | | | |
| Antrum | 73.3 | 45.8 | 27.9 | < 0.01 |
| Corpus | 9.5 | 8.0 | 4.5 | NS |

Figures are [a]medians or [b]per cent. The grading of gastritis was performed in accordance with the Sydney System with slight modifications as described elsewhere (Bayerdörffer et al, 1992; Stolte et al, 1995). NS, not significant ($P \geq 0.05$).

1). Focal atrophy was significantly more common in the antrum and corpus of carcinoma patients. Lymphoid follicles were detected with equal frequency in the antrum of carcinoma patients and control subjects, but significantly more frequently in the corpus of carcinoma patients. IM was equally present in the corpus of carcinoma patients and control subjects, but significantly higher in the antrum of carcinoma patients than in control subjects. On the basis of these findings the odds ratios were calculated for the relative risk of gastric carcinoma when the expression of a given parameter was either high grade or present at all. Expression of high-grade gastritis in the corpus means an 8.85-fold higher risk for gastric carcinoma, and high-grade activity in the corpus a 5.2-fold higher risk, whereas the expression of high-grade regenerative epithelium means a 13.1-fold higher risk for gastric carcinoma. Occurrence of focal atrophy or IM in the antrum means an 8-fold or 3.5-fold higher risk for gastric carcinoma respectively. The occurrence of lymphoid follicles in the corpus is associated with a 7.4-fold higher risk for gastric carcinoma.

### DISCUSSION

Evidence from epidemiological studies suggests a relative risk for subsequent development of gastric carcinoma of 3–6 in *H. pylori*-infected persons (Nomura et al, 1991; Parsonnet et al, 1991; Eurogast Study Group, 1993). The impracticability of preventive treatment of all *H. pylori*-infected individuals implies an urgent need to identify patients who are at increased risk for the development of gastric carcinoma. In addition to environmental, nutritional and sociocultural factors (Nomura et al, 1993; Sipponen, 1994), we hypothesize that the course and expression of *H. pylori* gastritis itself may contribute to the risk of gastric carcinoma.

A human model of gastric carcinogenesis proposed by Correa (1988) states that the loss of gastric mucosal glands, i.e. atrophy, and their replacement by intestinal-type epithelium (IM), might be a basic link in the chain of events leading to the development of gastric carcinoma. In the present study the frequency of IM was significantly higher in the antrum of gastric carcinoma patients, giving rise to a relative risk factor of 3.4. Many investigators consider IM to be a direct precursor of gastric carcinoma (Morson, 1955; Correa et al, 1990*a*; Craanen et al 1992), but the less frequent finding of IM in early gastric carcinoma (68%) compared with a higher frequency in advanced tumour stages (95% in our material) suggests that IM is a marker rather than a direct precursor in the natural history of gastric carcinoma. The results of a recent paper by Kimura et al (1993) and others (Shousha et al, 1993) also strongly support the hypothesis that IM is not a precursor lesion.

Another, no less important, interpretation of IM is that its presence also marks the influence of *H. pylori*-independent, mostly environmental or socioeconomic, factors on the risk of gastric carcinoma development. This is supported by data showing an 80% frequency of IM in biopsy material from two groups with different frequencies of *H. pylori* infection and different cancer risks (Correa et al, 1990*b*). This IM incidence is significantly higher than that in German patients, in whom we found only 28% in *H. pylori*-infected subjects, and only 5% in uninfected subjects (Bayerdörffer et al, 1992). Similar observations have been made by other investigators (Eidt and Stolte, 1994; Kuipers et al, 1995). However, as IM was more common in *H. pylori*-infected carcinoma patients than in infected non-carcinoma patients, and also more common in *H. pylori*-negative carcinoma patients than in uninfected non-carcinoma patients, it is very possible that IM may be a marker for an increased gastric carcinoma risk in *H. pylori*-infected as well as uninfected subjects.

Increased cell proliferation has been identified as an underlying mechanism for increased mutagenesis, and possibly initiates carcinogenesis (Ames and Gold, 1990; Eidt et al, 1995); it has also been thought to be a consequence of chronic *H. pylori* infection (Cahill et al, 1994; Correa et al, 1994). As measuring mucosal proliferation is complicated and time-consuming, replacement of the surface epithelium by regenerative epithelium (RE) can be assessed instead, because this parameter is closely correlated with the grade of epithelial proliferation (Stolte et al, 1995). In the present study, the presence of high-grade RE was found to be significantly higher in the corpus of gastric carcinoma patients, and the factor calculated for the associated relative risk for gastric carcinoma, namely 13, was the highest of all the parameters we investigated.

Of further importance for the pathogenesis of gastric cancer may be the fact that neutrophils that produce excessive amounts of reactive oxygen metabolites penetrate the epithelium at the bottom of the gastric pits and preferentially cluster in the regions where the stem cells are located (Davies et al, 1994). The activity of gastritis, which is a measure of neutrophil infiltration, was also significantly higher in the corpus mucosa of carcinoma patients in the present study, and a relative risk for gastric carcinoma of 5.3 was calculated.

Our data suggest that assessing the grade of gastritis in the corpus, the grade of activity in the corpus and the grade of regenerative epithelium in the corpus may be a valuable tool for identifying patients carrying an increased gastric carcinoma risk. In no other subgroup of *H. pylori*-infected patients, with the exception

© Cancer Research Campaign 1998

**Figure 1A–D** Comparison between grades of lymphocyte/plasma cell formation, neutrophil infiltration, regenerative epithelium and frequency of lymphoid follicles in the corpus mucosa of the 50 *H. pylori*-positive gastric carcinoma patients with regard to both histological subtypes (intest, intestinal; diff, diffuse) and controls. The statistical difference between the medians was calculated using the chi-square test. NS, not significant ($P \geq 0.05$). ●, $P < 0.01$

of some gastric ulcer patients, is *H. pylori* gastritis expressed in this particular manner (Meining et al, 1997). One might argue that a more severe expression of gastritis may be a consequence of advanced gastric carcinoma rather than a precursor condition; however, more than half of the tumours were found at lower stages, i.e. T1 or T2 (Table 1), and the biopsy specimens for grading of gastritis were taken a considerable distance away from the tumour. Furthermore, in a previous study of our group high-grade corpus gastritis was also found in 117 patients with early gastric cancer (stage T1) (Meining et al, 1998). Therefore, it appears more likely to us that severe corpus gastritis may precede gastric cancer rather than being a consequence of it. Severe corpus gastritis may be associated with impaired acid secretion capacity (Lee et al, 1995; Oi, 1995), and with focal atrophy and IM. In contrast, duodenal ulcer patients who have a very low gastric cancer risk (Hansson et al, 1996) show an antrum-predominant gastritis and have a higher gastric acid output (Lee et al, 1995). The differences in acid seceretion capacities and thus the type and extent of gastritis may be determined by the fundic–pyloric border as suggested by Oi (1995). One might therefore speculate that this most likely inherited factor may determine the expression of *H. pylori* gastritis before the development of gastric cancer.

In conclusion, our data suggest that high-grade gastritis or activity and high-grade expression of regenerative epithelium in the corpus mucosa may be associated with an increased risk for the development of gastric carcinoma. As these gastritis characteristics are expressed as high grade in only a small percentage of *H. pylori*-infected persons, they might serve as criteria for identifying patients for preventive treatment of *H. pylori* infection.

### ACKNOWLEDGEMENT

We are grateful to M Weber for his help during the study in collecting and analysing data.

### REFERENCES

Ames B and Gold LS (1990) Too many rodent carcinogens: mitogenesis increases mutagenesis. *Science* **249**: 970–971

Bayerdörffer E, Lehn N, Hatz R, Mannes GA, Oertel H, Sauerbruch T and Stolte M (1992) Difference in expression of *Helicobacter pylori* gastritis in antrum and body. *Gastroenterology* **106**: 1575–1582

Cahill RJ, Sant S, Beattie S, Hamilton H and O'Morain C (1994) *Helicobacter pylori* and increased epithelial proliferation: a risk factor for cancer. *Eur J Gastroenterol Hepatol* **6**: 1123–1127

Craanen ME, Dekker W, Blok P, Ferwerda J and Tytgat GNJ (1992) Intestinal metaplasia and *Helicobacter pylori:* an endoscopic bioptic study of the gastric antrum. *Gut* **33**: 16–20

Correa P (1988) A human model of gastric carcinogenesis. *Cancer Res* **48**: 3554–3560

Correa P, Haenszel W, Cuello C, Zavala D, Fontham E, Zarama G, Tannenbaum S, Collazos T and Ruiz B (1990*a*) Gastric precancerous process in a high risk population: cross sectional study. *Cancer Res* **50**: 4731–4736

Correa P, Fox J, Fontham E, Fontham DPH, Ruiz B and Lin Y (1990*b*) *Helicobacter pylori* and gastric carcinoma: serum antibody prevalence in populations with contrasting cancer risks. *Cancer* **66**: 2569–2574

Correa P (1991) Is gastric carcinoma an infectious disease? *N Engl J Med* **325**: 1170–1171

Correa P, Ruiz B, Shi T-Y, Janney A, Sobhan M, Torrado J and Hunter F (1994) *Helicobacter pylori* and nuclear organizer regions in the gastric antral mucosa. *Am J Clin Pathol* **101**: 656–660

Davies GR, Banatvala N, Collins CE, Sheaff MT, Abdi Y, Clements L and Rampton DS (1994) Relationship between infective load of *Helicobacter pylori* and reactive oxygen metabolite production in antral mucosa. *Scand J Gastroenterol* **29**: 419–424

Eidt S and Stolte M (1994) Prevalence of intestinal metaplasia in *Helicobacter pylori* gastritis. *Scand J Gastroenterol* **29**: 607–610

Eidt S, Eidt H and Stolte M (1995) Inflammatory reaction and epithelial proliferation of the gastric mucosa. A possible pathomechanism in cancerogenesis. *Pathologie* **16**: 192–196

Eurogast Study Group (1993) An international association between *Helicobacter pylori* infection and gastric cancer. *Lancet* **341**: 1359–1362

Hansson L-E, Nyren O, Hsing AW, Bergström R, Josefsson S, Chow W-H, Fraumeni JF and Adami H-O (1996) The risk of stomach cancer in patients with gastric or duodenal ulcer disease. *N Engl J Med* **335**: 242–249

Kimura K, Satoh K, Yoshida Y, Taniguchi Y, Ido K and Takemoto T (1993) Chronological extension of atrophic gastritis and intestinal metaplasia in normal Japanese. *Eur J Gastroenterol Hepatol* **5** (Suppl. 1): S85–S91

Kuipers EJ, Uyterlinde AM, Pena AS, Roosendaal R, Plas G, Nelis GF, Festen HPM and Meuwissen SGM (1995) Long-term sequelae of *Helicobacter pylori* gastritis. *Lancet* **345**: 1525–1528

Lauren P (1965) The two main histological types of gastric carcinoma: diffuse and so-called intestinal type carcinoma. An attempt at a histo-clinical classification. *Acta Pathol Microbiol Scand* (A) **64**: 31–39

Lee A, Dixon MF and Danon SJ (1995) Local acid production and *Helicobacter pylori*: a unifying hypothesis of gastroduodenal disease. *Eur J Gastroenterol Hepatol* **7**: 461–465

Meining A, Stolte M, Hatz R, Lehn N, Miehlke S, Morgner A and Bayerdörffer E (1997) Differing degree and distribution of gastritis in *Helicobacter pylori*-associated diseases. *Virch Arch* **431**: 11–15

Meining A, Stolte M, Müller P, Miehlke S, Lehn N, Hölzel D and Bayerdörffer E (1998) Gastric carcinoma risk index in patients infected with *Helicobacter pylori*. *Virch Arch* **432**: 311–314

Morson BC (1955) Carcinoma arising from areas of intestinal metaplasia in the gastric mucosa. *Br J Cancer* **9**: 377–385

Nomura A, Stemmermann GN, Chyou PH, Kato I, Perez-Perez GI and Blaser MJ (1991) *Helicobacter pylori* infection and gastric carcinoma among Japanese Americans in Hawaii. *N Engl J Med* **325**: 1132–1136

Nomura A and Stemmermann GN (1993) *Helicobacter pylori* and gastric cancer. *J Gastroenterol Hepatol* **8**: 294–303

Oi M (1995) Peptic ulcer and gastric acidity: a new look at an old aphorism, 'the higher the ulcer, the lower the acidity'. *J Gastroenterol* **30**: 122–127

Parsonnet J, Friedman GD, Vandersteen DP, Chang Y, Vogelman JH, Orentreich N and Sibley RK (1991) *Helicobacter pylori* infection and the riks of gastric carcinoma. *N Engl J Med* **325**: 1127–1131

Price A (1991) The Sydney System: histological division. *J Gastroenterol Hepatol* **6**: 209–222

Shousha S, Ei-Sherif AM, El-Guneid A, Arnaout AH and Murray-Lyon IM (1993) *Helicobacter pylori* and intestinal metaplasia: comparison between British and Yemeni patients. *Am J Gastroenterol* **88**: 1373–1376

Sipponen P (1994) Gastric cancer – a long-term consequence of *Helicobacter pylori* infection? *Scand J Gastroenterol* **29** (suppl. 201): 24–27

Stolte M, Stadelmann O, Bethke B and Burkard G (1995) Relationships between the degree of *Helicobacter pylori* colonisation and the degree and activity of gastritis, surface epithelial degeneration and mucus secretion. *Z Gastroenterol* **33**: 89–93

© Cancer Research Campaign 1998